# Comprehensive bile acid pool analysis during ex-vivo liver perfusion in a porcine model of ischemia-reperfusion injury

**DOI:** 10.1038/s41598-024-52504-7

**Published:** 2024-01-29

**Authors:** Guillaume Rossignol, Xavier Muller, Thomas Alexandre Brunet, Valeska Bidault, Valerie Hervieu, Yohann Clement, Sophie Ayciriex, Jean-Yves Mabrut, Arnaud Salvador, Kayvan Mohkam

**Affiliations:** 1 Department of General Surgery and Liver Transplantation, Croix Rousse University Hospital, Lyon, France; 2 Department of Pediatric Surgery and Liver Transplantation, Femme Mere Enfant University Hospital, Lyon, France; 3 The Cancer Research Center of Lyon, INSERM U1052, Lyon, France; 4 ED 340 BMIC, Claude Bernard Lyon 1 University, Villeurbanne, France; 5 Institute of Analytical Sciences, CNRS UMR 5280, Claude Bernard University Lyon 1, Villeurbanne, France; 6 Department of Pathology, Hospices Civils de Lyon, Claude Bernard Lyon 1 University, Villeurbanne, Lyon, France

**Keywords:** Liver, Experimental models of disease

## Abstract

Bile acids (BA) are key for liver regeneration and injury. This study aims at analyzing the changes in the BA pool induced by ischemia-reperfusion (IRI) and investigates the impact of hypothermic oxygenated perfusion (HOPE) on the BA pool compared to static cold storage (SCS). In a porcine model of IRI, liver grafts underwent 30 min of asystolic warm ischemia followed by 6 h of SCS (n = 6) ± 2 h of HOPE (n = 6) and 2 h of ex-situ warm reperfusion. The BA pool in bile samples was analyzed with liquid chromatography coupled with tandem mass spectrometry. We identified 16 BA and observed significant changes in response to ischemia-reperfusion, which were associated with both protective and injury mechanisms. Second, HOPE-treated liver grafts exhibited a more protective BA phenotype, characterized by a more hydrophilic BA pool compared to SCS. Key BA, such as GlycoCholic Acid, were identified and were associated with a decreased transaminase release and improved lactate clearance during reperfusion. Partial Least Square-Discriminant Analysis revealed a distinct injury profile for the HOPE group. In conclusion, the BA pool changes with liver graft IRI, and preservation with HOPE results in a protective BA phenotype compared to SCS.

## Introduction

Ischemia-reperfusion injury (IRI) impairs graft function recovery^1,2^ but also triggers bile duct injury^3^ in liver grafts after transplantation. Thus, investigating the impact of IRI on bile composition may allow to further refine the mechanisms of post-transplant bile duct injury^4,5^. In this context, a key target are bile acids (BA) which play an important role in various homeostatic metabolic processes in the liver^6^ through specific signaling pathways such as FXR or TGR5^7^. Indeed, BA have been shown to contribute to liver regeneration^8^, promote anti-inflammatory or pro-inflammatory responses and trigger liver injury as well as cholangiocyte injury^9,10^. Thereby, impairment of BA homeostasis by IRI may contribute to post-LT bile duct injury^11^ and the accumulation of “toxic” BA may be associated with hepatocyte and cholangiocyte injury^12,13^. A comprehensive analysis of BA associated with IRI is even more relevant in the current era of machine perfusion strategies, aiming to mitigate IRI, improving post-LT outcomes^14–16^ and allowing for viability assessment of liver grafts prior to LT^17,18^.

The primary objective of this study was therefore to conduct an extensive analysis of the BA pool using mass spectrometry in a porcine model of liver graft IRI. The specific aims of the study were to (a) investigate the alterations in the BA pool induced by IRI and (b) evaluate the potential impact of hypothermic oxygenated perfusion (HOPE) on the BA pool.

## Materials and methods

### Study aim

**Figure 1:**
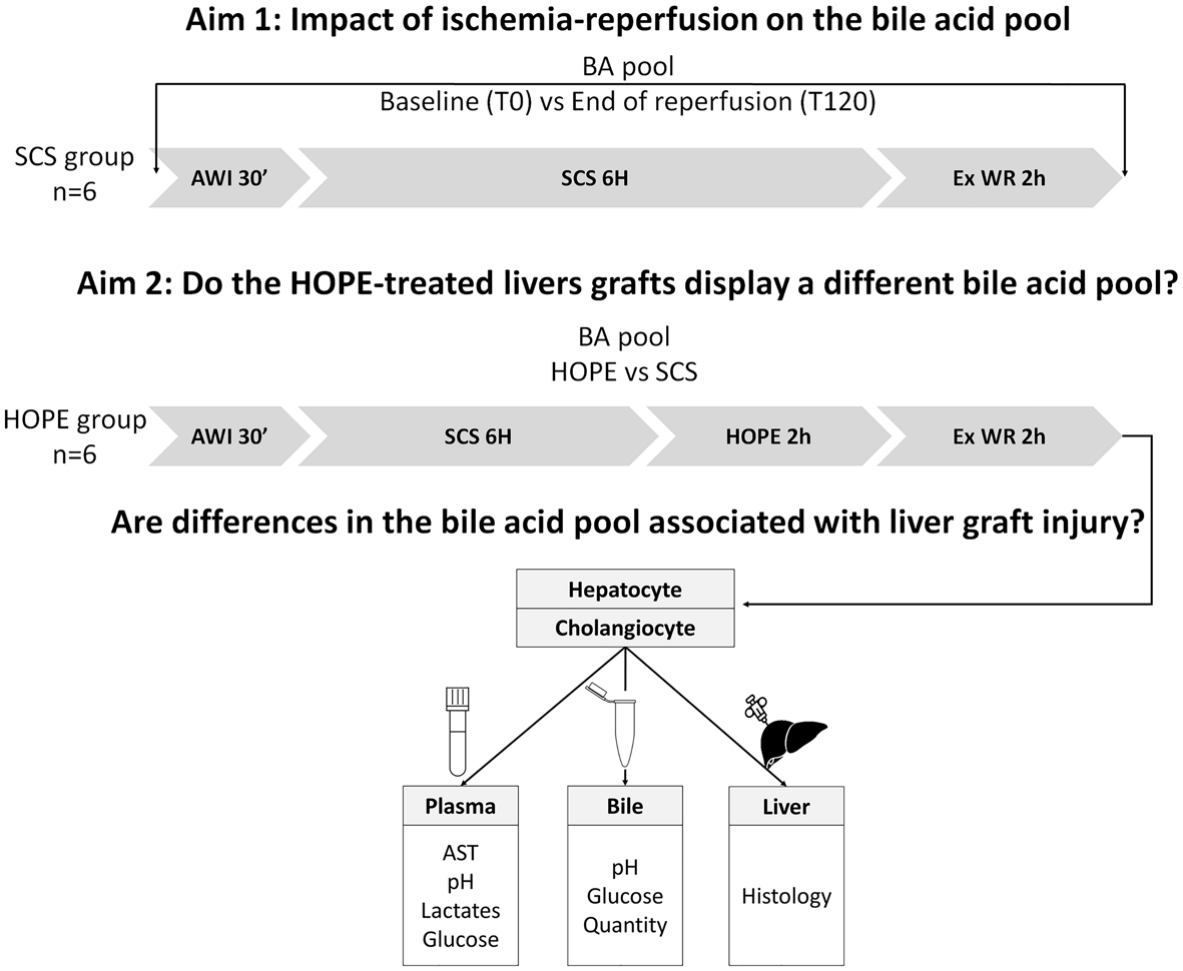
Study flow chart. SCS: Static Cold Storage, HOPE: Hypothermic Oxygenated Perfusion, AWI: Asystolic Warm Ischemia, Ex WR: Ex-situ Warm Reperfusion, BA: Bile Acids, AST: Aspartate aminotransferase.

First, we analyzed the changes in the bile acid pool induced by IRI in a porcine model of liver grafts with prolonged warm and static cold ischemia. Second, we investigated the impact of HOPE on the BA pool in comparison to static cold storage (SCS) and correlated these data with surrogate markers of hepatocyte and cholangiocyte IRI (Fig. 1).

### Study design

We used a previously reported porcine model of liver grafts to simulate early IRI^19,20^ and to determine the BA pool in bile samples after 2 h of ex-situ warm reperfusion in liver grafts subjected to SCS or SCS + HOPE (Fig. 1).

Briefly, liver grafts underwent 30 min of asystolic warm ischemia (AWI) prior to organ procurement followed by either static cold storage preservation for 6 h (n = 6, SCS group) or SCS + 2 h of end-ischemic HOPE (n = 6, HOPE group). The SCS group was used as control with expected higher IRI in comparison to the HOPE group^21^.

HOPE was performed ex-situ using the Liver Assist© device (XVIVO©, Sweden) through the portal vein only. The perfusion pressure was limited to 3 mmHg in the portal vein to reach a target portal flow ranging from 150 to 300 ml/min.

Finally, ex-situ warm reperfusion was performed at 37 °C for 2 h using the Liver Assist© device (XVIVO©, Sweden) to mimic liver transplantation and to focus on the early acute phase of IRI^22^. During reperfusion, no sodium bicarbonate, parenteral nutrition or glucose/insulin was added to avoid bias in evaluating graft function recovery.

### Ethics

This study is reported in accordance with the ARRIVE guidelines for reporting experiments involving animals. All animals received human care following the Guide for the Care and Use of Laboratory Animals (National Institutes of Health publication 86–23, revised in 1985).

The experimental protocol was approved by the Institutional Animal Experimentation Committee of Claude Bernard University Lyon 1 (CEEA-55A; project number DR2020-27).

### LC-MS/MS bile acid analysis

Bile samples were procured in the pig donor prior to liver procurement (baseline sample) and at the end of 2 h of warm reperfusion (last 10 min of reperfusion). All bile samples were snap-frozen and stored at − 80 °C. Prior to Liquid Chromatography coupled with tandem Mass Spectrometry (LC-MS/MS) analysis, methanol (MeOH) was added to bile samples with a ratio of 2.3/1 (70% bile, 30% MeOH). After centrifugation at 10000*g* at 4 °C during 10 min for protein precipitation and removal, the supernatant was collected and diluted at tenfold for LC-MS/MS analysis. LC-MS/MS analysis was conducted on a QTRAP^®^ 5500 LC-MS/MS system hybrid triple quadrupole/linear ion trap mass spectrometer (Sciex) equipped with a Turbo V™ ion source coupled with a 1290 series HPLC device (Agilent Technologies, Waldbronn, Germany). Chromatographic separation was performed by Reversed Phase Liquid Chromatography (RPLC) on a Kinetex F5 column (150 mm × 2.1 mm × 2.6 µm). MS analysis was configured in negative ionization mode using a spray voltage of − 4500 V. The nebulizer and the curtain gas flow were set at 40 and 50 psi respectively using nitrogen. The TurboV™ ion source was set at 450 °C with the auxiliary gas flow (nitrogen) set at 40 psi. Data acquisition was performed using Analyst 1.6.3 software^®^ (Sciex) and data processing with MultiQuant™ analysis software (vs 2.1.1., Sciex). The acquisition was done in a targeted fashion by Multiple Reaction Monitoring (MRM) and the method was developed and optimized on pure bile acid standards (BACSMLS, Sigma-Aldrich, France). Stability and reproducibility of LC-MS/MS experiments were monitored by QC samples and signal drift was corrected by Locally Estimated Scatterplot Smoothing (LOESS) using NormalizeMets package under R. Data sets obtained were then analyzed under R (v4.2.1, https://www.r-project.org), using both ropls (v1.28.2, https://bioconductor.org/packages/ropls) and mixomics package (v6.16.3, https://mixomics.org).

BA concentrations were expressed in percentage of the overall analyzed BA pool providing a more comprehensive perspective on variations in the bile acid phenotype in our study.

### Hepatocyte and cholangiocyte injury markers

We correlated the results obtained at the level of the BA pool to IRI markers at the hepatocyte and cholangiocyte level. Blood samples first underwent centrifugation at 1500 rpm for 10 min, and the obtained plasma was analyzed. Plasma levels of transaminase (Aspartate Amino Transferase, AST), pH, lactate and glucose were evaluated during ex-situ warm reperfusion. At the end of reperfusion, total bile production as well as bile pH and glucose were assessed. Based on previously published data^23,24^, those biochemical parameters reflect both hepatocyte and cholangiocyte damage during IRI.

In addition to these biochemical indicators, histological analysis of hepatocyte and cholangiocyte compartments was also performed. Liver and extrahepatic bile ducts biopsies were formalin fixed, paraffin embedded, and stained with HPS (hematoxylin, phloxine and saffron stain). A histological IRI assessment according to a standardized classification adapted from the UCLA classification and Suzuki classification was performed in a blinded manner by an expert pathologist^25,26^. In detail, IRI injury focused on apoptosis, ballooning, necrosis and neutrophilic infiltrate and were graded from 0 (none) to 4 (severe).

Both intrahepatic and extrahepatic bile ducts were identified using CK7 staining (Dako M7018) to evaluate IRI injury based on op den Dries et al.^27^ in a blinded manner by an expert pathologist. Histological analysis of bile duct injury focused on mural loss and mural necrosis graded from 0 (none) to 3 (severe) and loss of cell in peri-luminal and deep peri-biliary gland graded from 0 to 2 (severe) in both intrahepatic and extrahepatic bile ducts^28^.

### Statistical analysis

Continuous variables are expressed as median with interquartile range [IQR]. Continuous variables were compared using the non-parametric Mann–Whitney univariate test. Categorical variables were compared using the chi-square test or the Fisher’s exact test.

A descriptive and unsupervised approach using Principal Component Analysis (PCA)^29^ was used to evaluate sample distribution and separation among groups based on bile acid composition. Then, a supervised strategy by Partial Least Square Discriminant Analysis (PLS-DA)^30^ was performed to model and highlight differences between injury groups. R2Y values represents goodness-fit measure and Q2 values estimated the predictive ability of the model. The quality of the model was assessed by bootstrap resampling (*p* = 1000). In addition, variable importance in projection (VIP) scores derived from PLS-DA were used for the selection of significant BA. VIP values > 1 were used as variable selection criterion^31^. Of note, in multivariable analysis, the use of numerous data as independent variables is prone to a high degree of correlation between those variables. In this particular situation, PCA and PLS-DA allow for a reduction of this co-linearity, hence their application in this study^32^.

Variables associated with a *p*-value < 0.05 were considered statistically significant. Statistical analysis was performed using IBM SPSS Statistics for Windows (Version 26.0. Armonk, NY: IBM Corp) and GraphPad Prism (Version 8.0.0 for Windows, GraphPad Software, San Diego, California USA, www.graphpad.com).

## Results

### Baseline bile acid pool

In baseline bile samples prior to liver procurement (n = 12), we identified a total of 16 different bile acids, including both primary and secondary bile acids (Table S1). Primary BA constituted the main BA (56% [48–60]) with a majority of glyco and tauro-conjugated BA (respectively 39% [34–41] and 35% [31–43]). A detailed composition of the baseline BA pool is presented in Fig. S1. Of note, bile composition prior to organ procurement did not differ between HOPE and SCS groups as shown in Fig. S2. The identified baseline BA pool was compared to the BA pool at the end of 2 h of ex-situ warm reperfusion in two different preservation modalities, namely SCS and HOPE.

### Impact of ischemia–reperfusion on the bile acid pool

**Figure 2:**
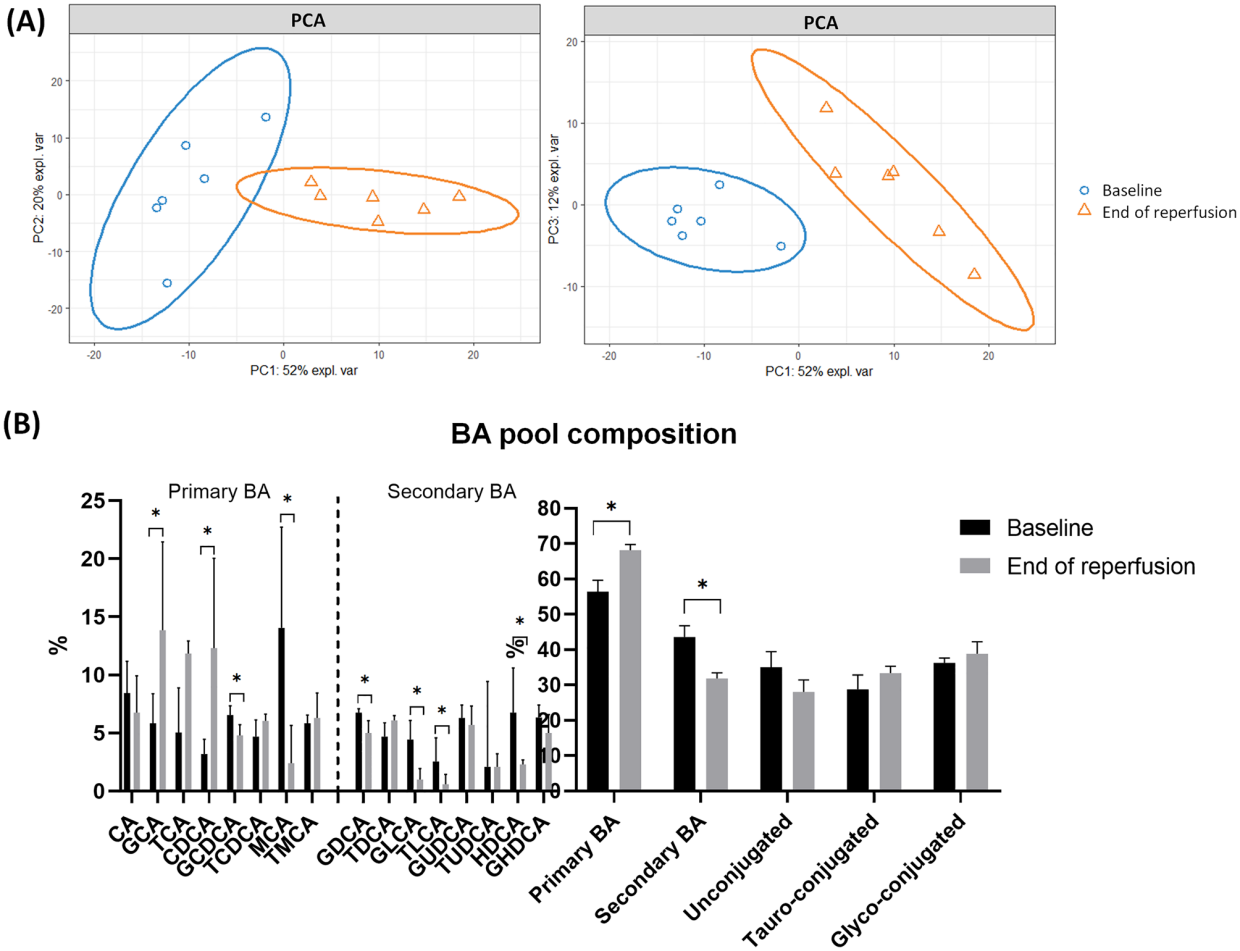
BA pool from baseline to the end of reperfusion. Panel (**A**): PCA displayed separation in BA pool composition. PCA scores with 52% of the variance explained on the first component, 20% on the second component and 12% on the third component. Panel (**B**): Detailed composition of BA pool from Baseline to the end of reperfusion. Variable are expressed as median [Inter quartile range, 25–75], non-parametric Mann–Whitney univariate test was used.

First, upon warm reperfusion after 6 h of SCS we observed a significant increase of primary BA (67.8% [64.5–72.2] vs 56.6% [49.1–58.9]; *p* = 0.02) with a significant increase of the Primary/Secondary BA ratio (P/S; *p* = 0.02) compared to the baseline (n = 6) (Fig. 2). We observed an increase in tauro/glyco-conjugated BA (*p* = 0.30 and *p* = 0.39 respectively) with a reduction in unconjugated BA (*p* = 0.28).

A detailed analysis of individual BA showed a significant increase in GlycoCholic Acid (GCA, *p* = 0.01) and ChenoDeoxyCholic Acid (CDCA, *p* = 0.01) whereas MuroCholic Acid (MCA, *p* = 0.006), GlycoChenoDeoxyCholic Acid (GCDCA, *p* = 0.02), GlycoDeoxyCholic Acid (GDCA, *p* = 0.04), GlycoLithoCholic Acid (GLCA, *p* = 0.004), TauroLithoCholic Acid (TLCA, *p* = 0.01) and HyoDeoxyCholic Acid (HDCA, *p* = 0.004) were significantly decreased (Fig. 2).

PCA presented in Fig. 2 exhibited a good separation between baseline and post reperfusion BA pool. PLS-DA (Fig. S2) was used to select individual BA of particular interest (VIP > 1) and exhibited good discrimination between baseline and post reperfusion BA pool (Q2Y = 0.801, R2X = 0.637, R2Y = 0.976) with a pR2Y < 0.05. In detail, GlycoLithoCholic Acid (GLCA), ChenoDeoxyCholic Acid (CDCA), MuroCholic Acid (MCA), HyoDeoxyCholic Acid (HDCA), GlycoChenoDeoxyCholic Acid (GCDCA), GlycoCholic Acid (GCA) and TauroLithoCholic Acid (TLCA) were the main BA involved in overall BA pool changes (Table S2).

### Do HOPE-treated liver grafts display a different bile acid pool?

**Figure 3:**
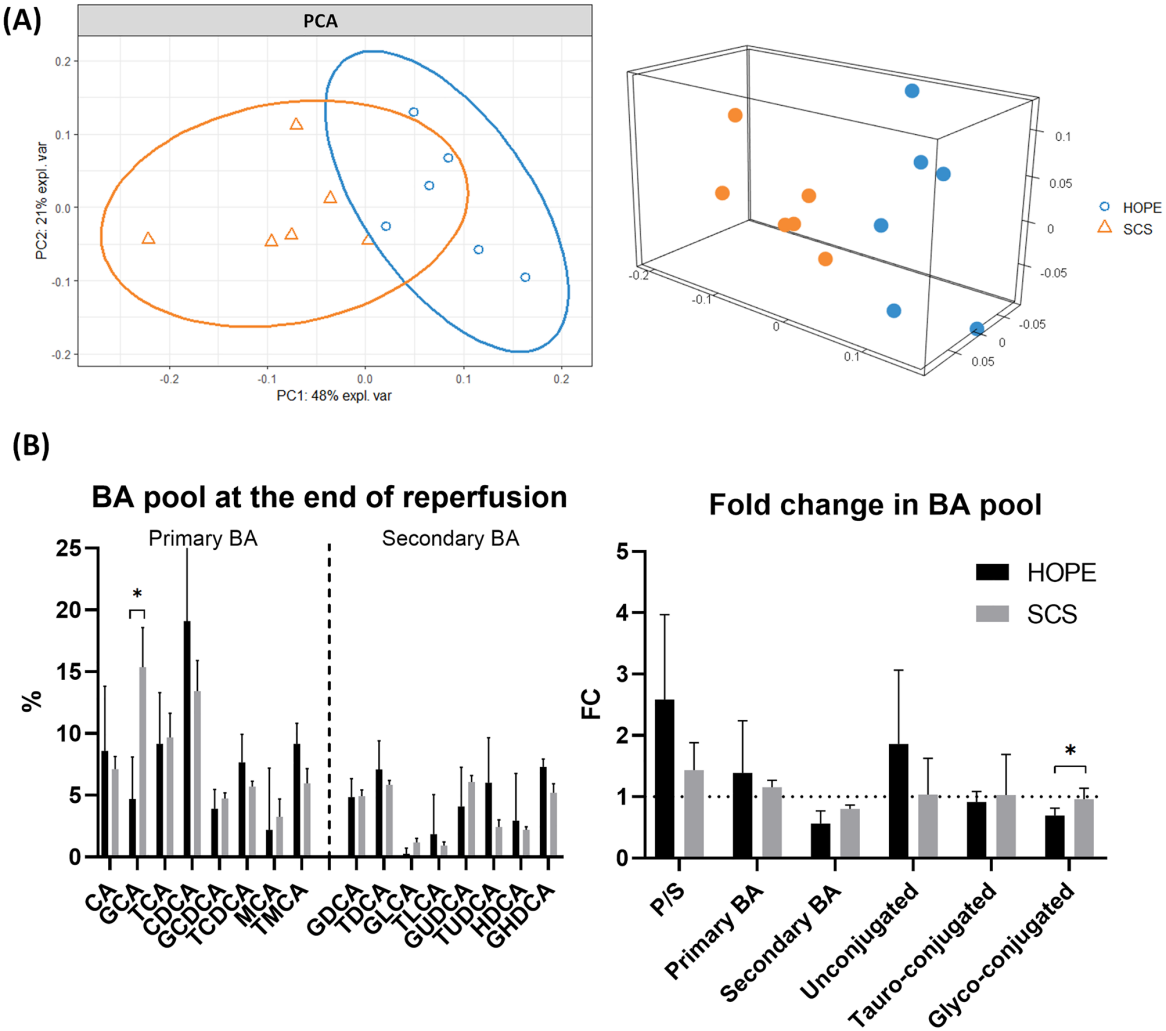
BA pool at the end of reperfusion in both groups. Panel (**A**): PCA displayed separation in BA pool composition between SCS and HOPE. PCA scores with 48% of the variance explained on the first component, 21% on the second component. Panel (**B**): Detailed BA pool at the end of reperfusion in both groups. Variable are expressed as median [Inter quartile range, 25–75], non-parametric Mann–Whitney univariate test was used. SCS: Static Cold Storage, HOPE: Hypothermic Oxygenated Perfusion, BA: Bile Acids, FC: Fold Change, P/S: Primary/Secondary ratio.

In comparison to SCS, HOPE-treated livers showed significantly lower GCA (*p* = 0.01) with higher levels of TauroMuroCholic Acid (TMCA, *p* = 0.15), TauroDeoxyCholic Acid (TDCA, *p* = 0.15) and TauroChenoDeoxyCholic Acid (TCDCA, *p* = 0.11) upon warm reperfusion (Table 1, Fig. 3). The proportion of glyco-conjugated BA was significantly lower in the HOPE group (23% [20–37] vs 37% [32–47], *p* = 0.01) with a higher P/S ratio (*p* = 0.10).

**Table 1:** BA pool at the end of reperfusion in HOPE and SCS groups.

Bile Acids	HOPE	SCS	*p* value	VIP (PLS-DA)
CA	8.6% [5.0–13.3]	6.8% [5.8–9.9]	0.63	–
GCA	4.7% [4.2–7.4]	13.9% [9.2–19.0]	0.01	1.27
TCA	9.1% [3.8–12.2]	11.8% [5.8–12.5]	0.63	1.71
CDCA	19.1% [13.5–29.4]	12.3% [11.5–19.9]	0.20	1.73
GCDCA	3.9% [3.4–5.3]	4.8% [3.8–5.6]	0.42	1.31
TCDCA	7.7% [6.1–9.5]	6.0% [4.8–6.6]	0.11	–
MCA	2.2% [0.5–6.7]	2.4% [0.5–4.3]	0.87	–
TMCA	9.2% [6.2–10.5]	6.3% [5.3–8.3]	0.15	–
GDCA	4.9% [3.5–6.0]	5.0% [4.2–6.0]	1.00	–
TDCA	7.1% [6.2–8.9]	6.1% [5.3–6.4]	0.15	–
GLCA	0.3% [0.2–0.3]	1.0% [0.6–1.8]	0.20	–
TLCA	1.9% [0.6–3.4]	0.6% [0.6–1.2]	0.26	–
GUDCA	4.1% [3.6–6.1]	5.7% [5.6–7.1]	0.20	–
TUDCA	6.0% [1.4–7.6]	2.1% [1.6–2.6]	0.34	1.08
HDCA	3.0% [1.8–5.6]	2.3% [1.5–2.6]	0.42	–
GHDCA	7.3% [6.0–7.9]	5.0% [4.2–6.1]	0.34	–

SCS: Static Cold Storage, HOPE: Hypothermic Oxygenated Perfusion, VIP: Variable Importance Projection Variable are expressed as median [Inter quartile range, 25–75].

When focusing on fold changes (FC) from the baseline to the end of reperfusion, we observed a significant decrease of glyco-conjugated BA in the HOPE group compared to SCS (FC: 0.7 vs 0.9, *p* = 0.02; Fig. 3) and an increase of unconjugated BA (1.8 vs 1.1, *p* = 0.06). We also observed higher P/S ratio in the HOPE group (2.6 vs 1.4, *p* = 0.08) with a higher proportion of primary BA (1.4 vs 1.1, *p* = 0.10) and less secondary BA (0.5 vs 0.8 *p* = 0.08).

PCA based on the BA pool at the end of reperfusion exhibited a separation between the two groups as shown in Fig. 3. PLS-DA was used to identify BA of particular interest (VIP > 1). ChenoDeoxyCholic Acid (CDCA), TauroCholic Acid (TCA), GlycoChenoDeoxyCholic Acid (GCDCA), GlycoCholic Acid (GCA) and TauroUrsoDeoxyCholic Acid (TUDCA), were the main BA involved in group discrimination (Table 1).

### Are differences in the bile acid pool associated with liver graft injury?

We observed a positive correlation between GlycoCholic Acid (GCA) levels and biochemical injury markers such as AST or Lactate (R spearman 0.6; *p* = 0.04 and 0.62; *p* = 0.03 respectively).

**Figure 4:**
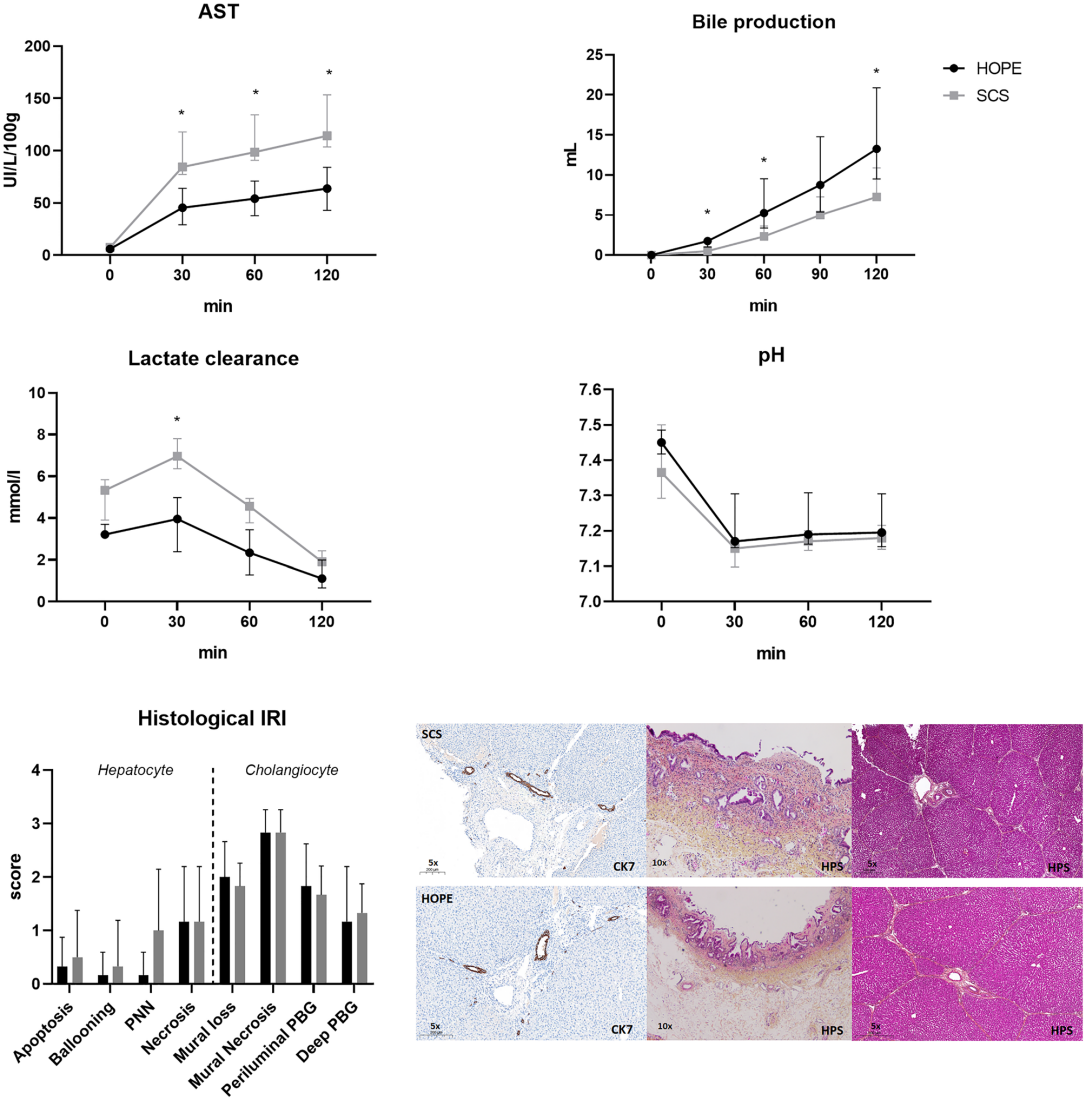
Biochemical injury markers and histological analysis comparing HOPE to SCS. Variable are expressed as median [Inter quartile range, 25–75], non-parametric Mann–Whitney univariate test was used. SCS: Static Cold Storage, HOPE: Hypothermic Oxygenated Perfusion, AST: Aspartate aminotransferase, IRI: Ischemia–Reperfusion Injury, PNN: Neutrophilic infiltrate, PBG: Peri-Biliary Gland, HPS: Hematoxylin, Phloxine and saffron stain.

In addition, the BA phenotype observed in the HOPE group was associated with significantly lower transaminase release (63 UI/L/100 g [43–77] vs 114 [105–132], *p* = 0.004) and a significant increase in cumulative bile production (12.5 ml [9–20] vs 7.5 [4–10], *p* = 0.04) compared to SCS. Lactate clearance and pH normalization were comparable between both groups during reperfusion with however less lactate release and a higher pH in the HOPE group (3.96 vs 6.96, *p* = 0.004 and 7.17 vs 7.15, *p* = 0.02 respectively, Fig. 4). We also observed a higher bile pH (7.47 [7.43–7.6] vs 7.29 [7.1–7.5]; *p* = 0.13] and a significant lower bile glucose (13.6 mmol/L [12.2–16.5] vs 22.1 [18.09–27.7]; *p* = 0.04] in the HOPE group.

We did not observe any difference in histological median hepatocyte injury score between HOPE and SCS groups as shown in Fig. 4 (1.5 vs 2.5; *p* = 0.35) as well as in median histological bile duct injury score (7.5 vs 8 points for HOPE and SCS respectively, *p* = 0.88; Fig. 4).

**Figure 5:**
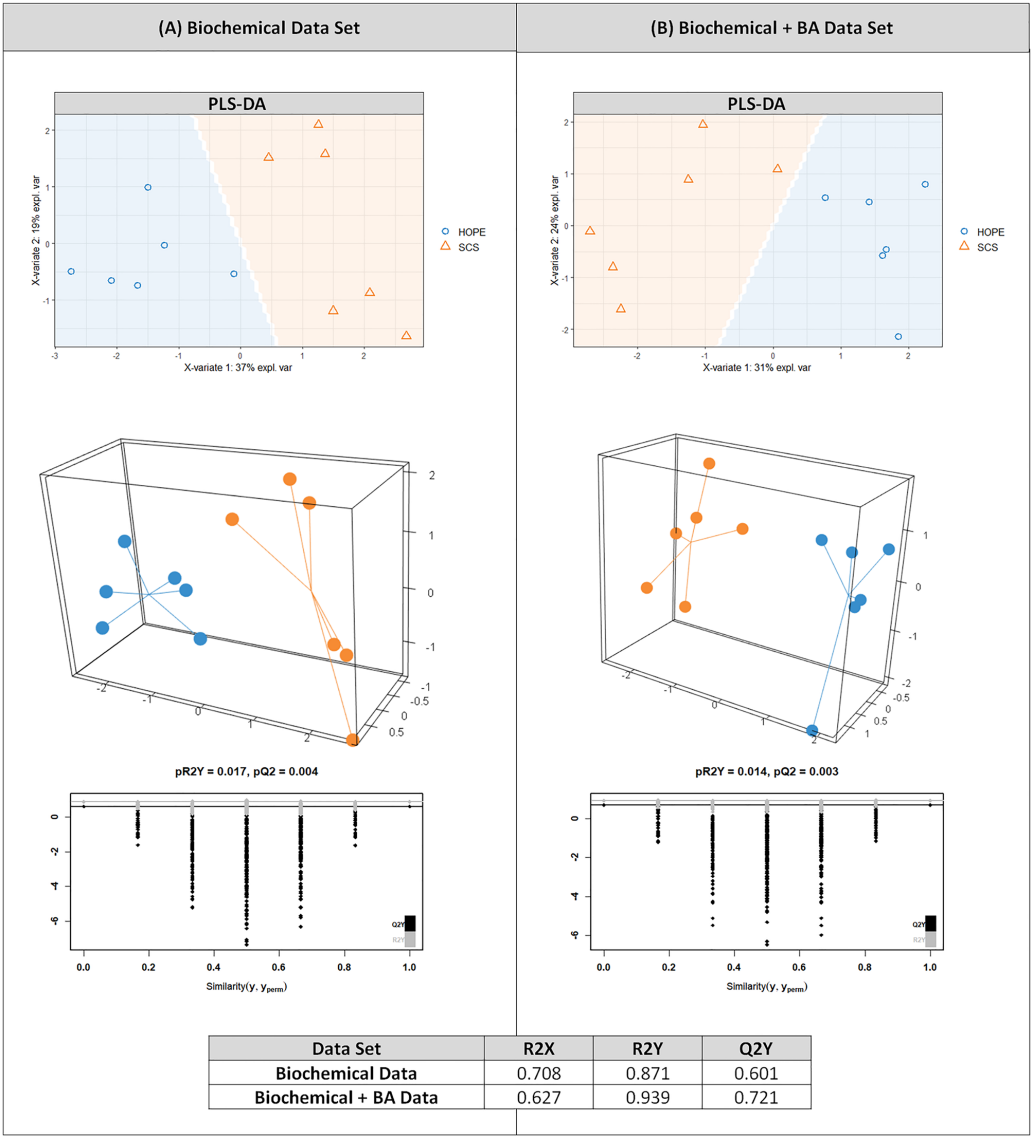
PLS-DA based on biochemical injury markers and BA pool in HOPE and SCS group. R2 values represents goodness-fit-measure and Q2 values estimated the predictive ability of the model. Panel (**A**) exhibits PLS-DA analysis based on biochemical data alone, 37% of the variance explained on the first component, 19% on the second component. Panel (**B**) exhibits PLS-DA analysis based on biochemical data and BA pool 31% of the variance explained on the first component, 24% on the second component. SCS: Static Cold Storage, HOPE: Hypothermic Oxygenated Perfusion, BA: Bile Acids.

Overall, the HOPE group exhibited a distinct IRI profile compared to SCS based on biochemical data ± BA pool composition as shown by PCA (Fig. S3). As shown in Fig. 5, PLS-DA analysis based on biochemistry alone ± BA pool composition allowed for a good discrimination among groups (Q2Y = 0.601 and Q2Y = 0.721 respectively with a pR2Y < 0.05).

## Discussion

In this study using a porcine model of liver graft ischemia-reperfusion injury, we conducted a comprehensive analysis of the bile acid pool following warm ex-situ reperfusion using LC–MS/MS analysis. First, we identified 16 BA of which 7 exhibited significant changes after IRI namely GlycoLithoCholic Acid (GLCA), ChenoDeoxyCholic Acid (CDCA), MuroCholic Acid (MCA), HyoDeoxyCholic Acid (HDCA), GlycoChenoDeoxyCholic Acid (GCDCA), GlycoCholic Acid (GCA) and TauroLithoCholic Acid (TLCA). Second, we found that HOPE-treated liver grafts exhibited a significant decrease in glyco-conjugated BA along with a more hydrophilic BA pool compared to SCS. The HOPE BA phenotype was associated with a decreased transaminase release and improved lactate clearance upon reperfusion resulting in a distinct liver graft injury profile compared to SCS. Changes in GlycoCholic acid (GCA) and to a lesser extent ChenoDeoxyCholic Acid (CDCA), TauroCholic Acid (TCA), GlycoChenoDeoxyCholic Acid (GCDCA) and TauroUrsoDeoxyCholic Acid (TUDCA) allowed to establish specific BA phenotypes for SCS and HOPE-treated liver grafts.

Investigating bile duct injury mechanisms^12,33^ is a key challenge with the aim of promoting bile duct integrity^16,34^ and enable bile duct viability assessment in liver grafts prior to LT^5,35^. At the level of biliary tree, the IRI cascade is initiated by ATP depletion and mitochondrial succinate accumulation^36^ during SCS. These early events impair BA secretion and reabsorption in both hepatocytes and cholangiocytes. Upon warm reperfusion in the recipient, ROS (reactive oxygen species) and DAMPs (damaged-associated molecular patterns) release, the accumulation of toxic hydrophobic BA^11^ associated with a damage bicarbonate umbrella^37^ trigger cholangiocellular injury. Besides those early IRI mechanisms, lack of regeneration of damaged peribiliary glands^27^ also leads to the loss of bile duct integrity hence the occurrence of ischemic type biliary lesions. In contrast, preservation of liver grafts with HOPE has been shown to protect from early IRI events by allowing energy recovery prior to reperfusion and reducing early ROS and DAMPs release after LT^38^. HOPE also improves cellular regeneration and BA secretion, thus decreasing the occurrence of ischemic type biliary lesions^5,16^. Besides the aforementioned indicators of IRI, BA have also been shown to be major determinants in the liver injury/protection balance^8^. For instance, bile salt secretion relative to phospholipid concentration has been proposed as a surrogate marker for assessing bile duct ischemic injury^39,40^. However to date, BA remain seldomly studied in the setting of IRI and liver graft preservation^5,11^. The aim of this study was thus to identify changes in BA pool induced by liver graft IRI and analyze the impact of different graft preservation strategies.

First, we attempted to characterize the impact of IRI on the BA pool of liver grafts. We identified 16 different BA by LC-MS/MS analysis which underwent significant changes from baseline to the end of the early reperfusion phase. This observation was further supported by PCA and PLS-DA analysis which both showed a clear separation between the BA pool at the 2 different time points. After 2 h of warm ex-situ reperfusion we indeed observed a significant increase of primary BA (*p* = 0.02), with a switch toward a more hydrophilic BA pool. Primary BA are known to be more hydrophilic BA thereby being more protective against liver injury^7^. On the other hand, BA overload or a shift toward a more hydrophobic BA pool (reflected by the primary/secondary BA ratio) may promote liver injury^7,8^. In detail, MuroCholic Acid (MCA) (*p* = 0.006) and GlycoChenoDeoxyCholic Acid (GCDCA) (*p* = 0.002) were significantly decreased at the end of reperfusion. MCA is known to be a FXR antagonist potentially impairing liver regeneration and the adaptive response to liver injury^41^. GCDCA has been associated with hepatocyte cell death and oxidative stress^42^. Consistent with the increase of ChenoDeoxyCholic Acid (CDCA) (*p* = 0.01) and the decrease of GlycoDeoxyCholic Acid (GDCA) (*p* = 0.04) in our study, these findings suggest a protective response from these livers when expose to IRI, in an attempt to mitigate BA mediated injury^43^. Besides those protective mechanisms, we observed an increase of GlycoCholic Acid (GCA) and a decrease of Tauro/Glyco-conjugated LithoCholic Acid, which are indicative of a higher degree of liver damage^44^. Finally, HyoDeoxyCholic acid (HDCA) (*p* = 0.004) was significantly increase at the end of reperfusion. HDCA has been linked to glucose homeostasis^45^ and bile glucose serves as a known surrogate marker for bile duct injury during normothermic perfusion^23^, thereby suggesting that HDCA may be more direct marker of bile duct viability. Altogether, these data suggest that the BA phenotype relates to IRI, demonstrating significant changes in line with the extent of liver injury as expressed by GlycoCholic Acid (GCA) or uncovering the protective adaptive response of liver grafts to mitigate liver IRI.

Second, we focused on the impact of two different preservation strategies on the BA pool by comparing the BA phenotype in liver grafts undergoing SCS versus HOPE. PCA revealed a clear separation between the HOPE and SCS groups based on their respective BA phenotype (Fig. 3). Overall, HOPE exhibited a more hydrophilic thus protective BA pool^7,8^ compare to SCS. HOPE indeed demonstrated a higher P/S ratio (*p* = 0.08) and a significant reduction in hydrophobic glyco-conjugated BA (*p* = 0.02) which are associated with the progression of liver injury^46^. In detail, we identified 5 specific BA that allowed to differentiate HOPE and SCS preserved liver grafts, namely GlycoCholic acid (GCA), ChenoDeoxyCholic Acid (CDCA), TauroCholic Acid (TCA), GlycoChenoDeoxyCholic Acid (GCDCA) and TauroUrsoDeoxyCholic Acid (TUDCA). The main discriminative BA was GlycoCholic Acid (GCA) which was significantly lower in the HOPE group (*p* = 0.03) compared to SCS and has been shown to be associated with a higher degree of liver damage^44,47^. In addition, levels of TauroCholic Acid (TCA) and GlycoChenoDeoxyCholic Acid (GCDCA) were both decreased in the HOPE group (*p* = 0.63 and *p* = 0.42 respectively) which points to less severe liver injury^44,48^. On the other hand, TauroUrsoDeoxyCholic Acid (TUDCA) was increased in the HOPE group (*p* = 0.58). This BA is involved in liver protective mechanisms as it decreases hepatic toxicity, inhibit pro-inflammatory signaling and mitigates cholangiocellular injury^49^. Altogether, the presented data suggest that HOPE-treated livers exhibited an overall more protective BA pool with GlycoCholic Acid being the main discriminative BA between SCS and HOPE in our study.

Third, we investigated whether the observed differences in BA phenotype were also associated with less severe IRI based on hepatocyte and cholangiocyte injury markers. Our findings demonstrated that HOPE-treated livers exhibited less severe IRI in univariable analysis consistent with previously published data^21^. We observed a significant reduction in transaminase release (63 UI/L/100 g vs 114, *p* = 0.004), improved lactate clearance (*p* = 0.04) and pH normalization (*p* = 0.02). Besides, PCA revealed a separation between HOPE and SCS groups based on these biochemical data, with a robust discrimination observed in PLS-DA (Q2Y > 0.5; Q2Y = 0.601; R2X = 0.708; R2Y = 0.871; Fig. S3). Moreover, when focusing on the key BA identified in PLS-DA, we found that GlycoCholic Acid positively correlated with transaminase release or lactate clearance (R spearman 0.6; *p* = 0.04 and 0.62; *p* = 0.03 respectively). These findings suggest that the changes observed in the HOPE BA phenotype correlated with biochemical markers of cellular injury in our study. In addition to GlycoCholic Acid (GCA), adding selected BA as IRI markers, improved the predictive ability of the PLS-DA model (Q2Y = 0.721, R2X = 0.627, pR2Y < 0.05) resulting in an improved discrimination between the HOPE and SCS group. These results suggest that BA phenotype may provide additional valuable information to refine liver IRI assessment. To further support this assumption, future clinical studies should correlate BA pool during ex-situ normothermic perfusion to relevant clinical endpoints such as post-reperfusion syndrome, ischemic type biliary lesions and graft loss.

Our study was designed as a proof-of-concept study aiming at evaluating the impact IRI and HOPE on BA homeostasis and has thus several limitations primarily the limited sample size. Second, we focused on the early phase of IRI^22^ which is the key trigger for downstream activation of the liver inflammasome. Thus, we were not able to draw any conclusions on long-term events and clinically relevant complications. However, the identified key BA in IRI pave the way for a rapid translational application. Third, it is important to acknowledge that BA physiology also involves the gut-liver axis which is not included in our isolated ex-situ reperfusion model^50^. This does however not preclude from analyzing bile composition and provide valuable insights as it takes into account both BA uptake and formation by the hepatocyte and the cholangiocyte^11,13^. In addition, ex-situ machine perfusion as viability assessment platform is also performed in the absence of a gut-liver axis, highlighting the translational potential of our data. Fourth, while BA composition has been shown to be comparable between tissue and bile, liver tissue biopsy may provide additional useful information regarding BA shifts and should be explored in future studies^51^. Finally, we performed a quantitative analysis of the BA pool which did not allow for a precise determination of single BA concentrations and their respective variations. The LC-MS/MS method was developed and optimized on pure bile acid standards (BACSMLS, Sigma-Aldrich, France) aiming at performing an exhaustive analysis of 40 BA. We observed 16 BA, and their respective values were expressed as percentages to facilitate understanding and result comparison. However, these percentages should not be used as reference values for future studies. While focusing on specific BA may be of interest from a mechanistic standpoint, the overall BA phenotype may offer more informative insights for assessing IRI profile compared to individual BA^8^.

In conclusion, in a porcine model of liver ischemia-reperfusion injury, we successfully identified 16 BA in bile samples after 2 h of ex-situ warm reperfusion. We observed significant changes in the BA pool induced by liver graft IRI, reflecting both the liver protective adaptive response and the extent of liver injury. Second, HOPE-treated liver grafts exhibited a more protective BA phenotype characterized by a shift towards a more hydrophilic BA pool and a significant reduction of GlycoCholic Acid (GCA). Notably, The HOPE BA phenotype was associated with a lower overall liver graft IRI profile compared to SCS. Future studies must validate the use of BA phenotype as a liver graft injury marker by correlating the latter to relevant clinical outcomes. Another future application of the presented data is the analysis of BA homeostasis during ex-situ machine perfusion to refine graft viability assessment prior to LT.

### Supplementary Information

Supplementary Information.

## Data Availability

The datasets generated and/or analysed during the current study are not publicly available but are available from the corresponding author on reasonable request.
