# Supplementary material for: Comprehensive bile acid pool analysis during ex-vivo liver perfusion in a porcine model of ischemia-reperfusion injury
Source: Sci Rep. 2024 Jan 29;14:2384. doi: 10.1038/s41598-024-52504-7 (PMC10824768; doi:10.1038/s41598-024-52504-7)

SUPPLEMENTARY INFORMATION

Tables:

Table S1: LC/MS-MS analysis of Bile Acids

BA : Bile Acid ; Q1-Q3 : Mass filter ; RT : Retention Time

Bile Acids				Q1	Q3	RT
**CA**	Cholic Acid	Primary BA	Unconj	407.2	407.2	24.623
**GCA**	Glyco-Cholic Acid		G-conj	464.328	464.285	23.25
**TCA**	Tauro-Cholic Acid		T-conj	514.379	80	23.19
**CDCA**	ChenoDeoxyCholic Acid		Unconj	391.112	391.112	25.188
**GCDCA**	Glyco-ChenoDeoxyCholic Acid		G-conj	448.404	386.2	22.45
**TCDCA**	Tauro-ChenoDeoxyCholic Acid		T-conj	498.383	80	25.77
**MCA**	MuriCholic Acid		Unconj	407.237	407.229	24.11
**TMCA**	Tauro-MuriCholic Acid		T-conj	514.212	513.692	20.5
**GDCA**	Glyco-DeoxyCholic Acid	Secondary BA	G-conj	448	74.2	23.3
**TDCA**	Tauro-DeoxyCholic Acid		T-conj	498.279	497.857	26.06
**GLCA**	Glyco-LithoCholic Acid		G-conj	432.322	388.3	28.82
**TLCA**	Tauro-LithoCholic Acid		T-conj	482.47	481.892	28.42
**GUDCA**	Glyco-UrsoDeoxyCholic Acid		G-conj	448.2	74	26.34
**TUDCA**	Tauro-UrsoDeoxyCholic Acid		T-conj	498.2	80	21.13
**HDCA**	HyoDeoxyCholic Acid		Unconj	391.2	391.2	23.49
**GHDCA**	Glyco-HyoDeoxyCholic Acid		G-conj	448.2	74	26.04

Table S2: BA pool at baseline and at the end of reperfusion in the SCS group

VIP: Variable Importance Projection

Variable are expressed as median [Inter quartile range, 25-75]

Bile Acids	Baseline	End of reperfusion	p value	VIP (PLS-DA)
**CA**	8.45% [5.6-10.5]	6.75% [5.8-9.9]	0.59	-
**GCA**	5.85% [4.7-7.7]	13.85% [9.2-19]	0.02	1.18
**TCA**	5.05% [4.7-8.4]	11.85% [5.8-12.5]	0.13	-
**CDCA**	3.2% [2.1-4.3]	12.3% [11.5-19.9]	0.01	1.41
**GCDCA**	6.55% [5.7-7.2]	4.8% [3.8-5.6]	0.02	1.24
**TCDCA**	4.7% [3.5-5.8]	6.05% [4.8-6.6]	0.24	-
**MCA**	14.05% [10.5-21.1]	2.4% [0.5-4.3]	0.004	1.32
**TMCA**	5.85% [3.4-6.1]	6.3% [5.3-8.3]	0.39	-
**GDCA**	6.75% [5.7-6.8]	5% [4.2-6]	0.04	-
**TDCA**	4.7% [3.1-5.7]	6.1% [5.3-6.4]	0.13	-
**GLCA**	4.45% [3.2-5.8]	1% [0.6-1.8]	0.002	1.44
**TLCA**	2.55% [2.2-3.9]	0.6% [0.6-1.2]	0.01	1.16
**GUDCA**	6.3% [5.3-7.3]	5.7% [5.6-7.1]	0.82	-
**TUDCA**	2.1% [1.5-8.8]	2.1% [1.6-2.6]	1.00	-
**HDCA**	6.75% [5.3-8.8]	2.3% [1.5-2.6]	0.002	1.28
**GHDCA**	6.35% [5-7.4]	5% [4.2-6.1]	0.49	-

Figures:

Figure S1: Baseline BA pool

Variable are expressed as median [Inter quartile range, 25-75]

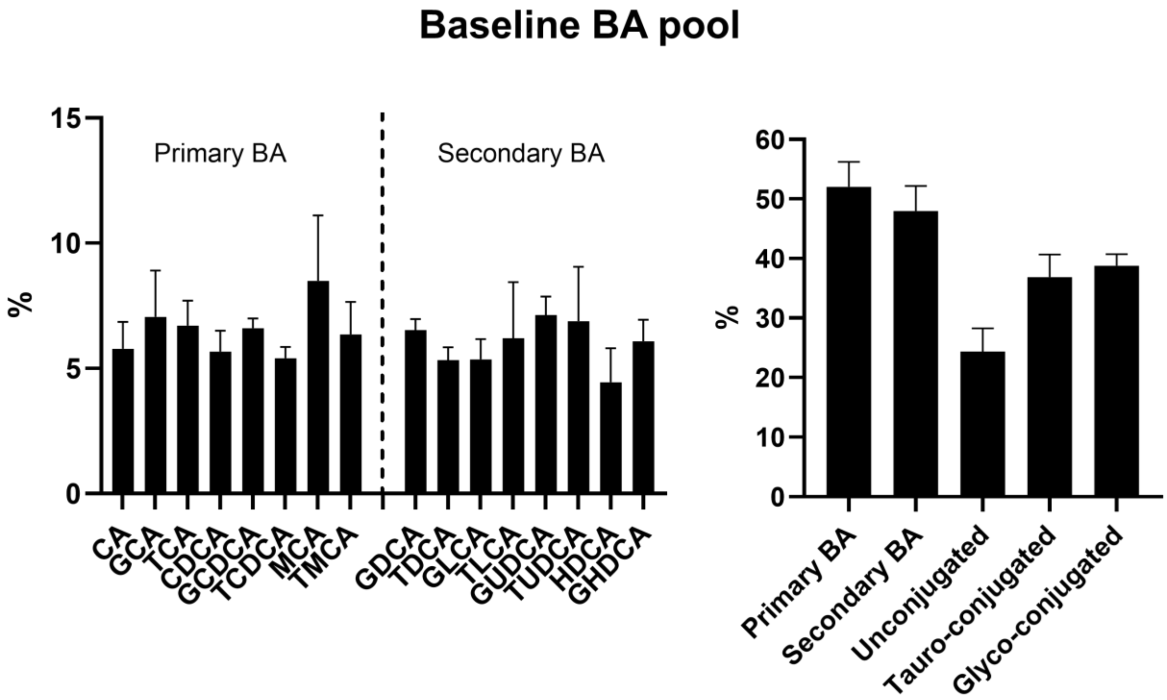

Figure S2: Baseline BA pool in HOPE and SCS

Panel A: PCA showed similar composition of BA in both groups. PCA scores with 73% of the variance explained on the first component, 14% on the second component and 6% on the third component.

Panel B: Detailed composition between HOPE and SCS at T0

Variable are expressed as median [Inter quartile range, 25-75]

SCS: Static Cold Storage, HOPE: Hypothermic Oxygenated Perfusion

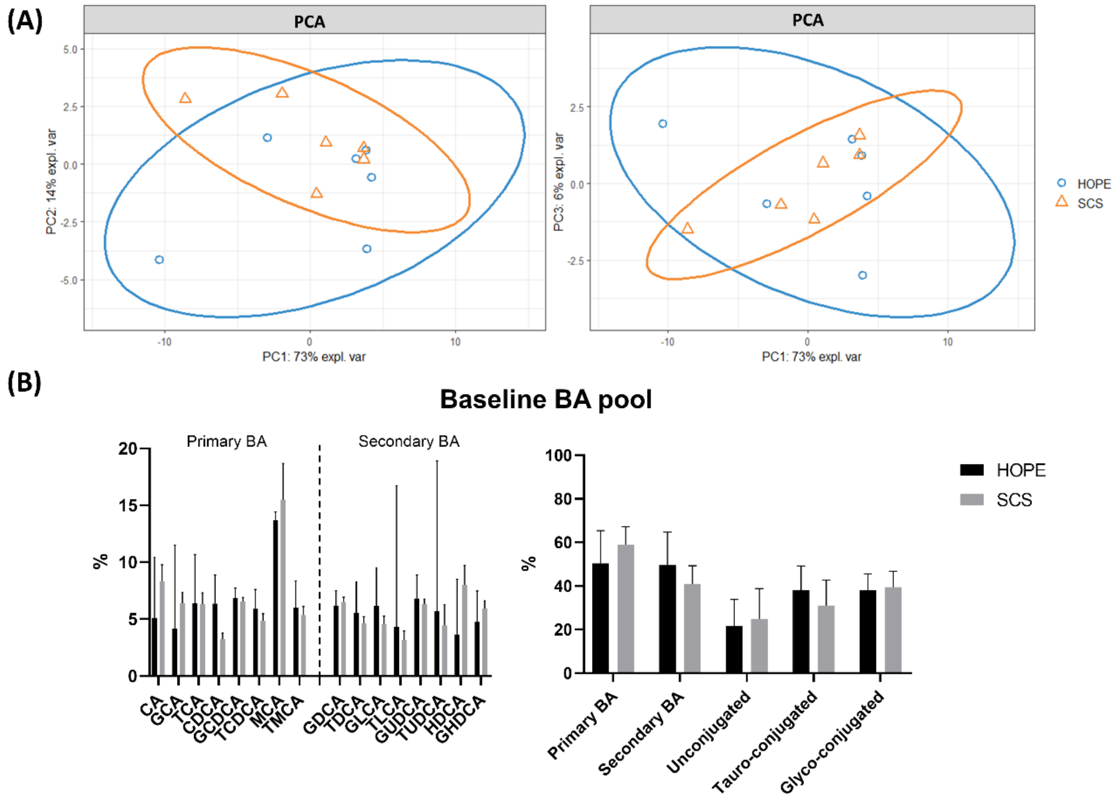

Figure S3: PLS-DA based on BA pool at baseline and the end of reperfusion.

R2 values represents goodness-fit-measure and Q2 values estimated the predictive ability of the model.

PLS-DA analysis showed a different BA pool with 33% of the variance explained on the first component, 19% on the second component.

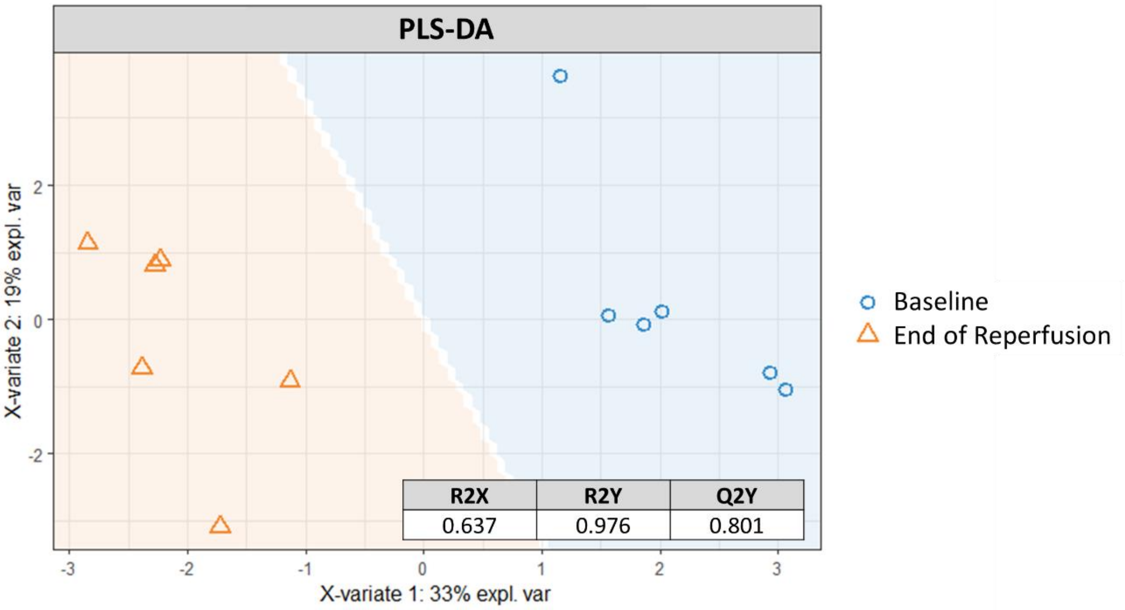
PLS-DA
X-variate 1: 33% expl. var
X-variate 2: 19% expl. var
Baseline
End of Reperfusion
R2X 0.637
R2Y 0.976
Q2Y 0.801

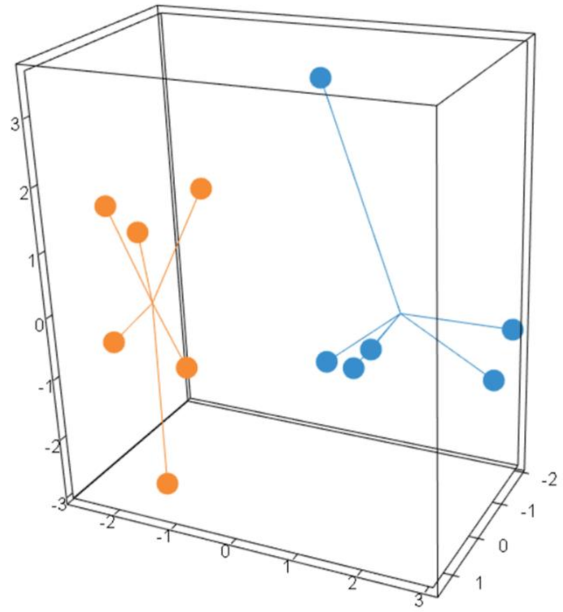

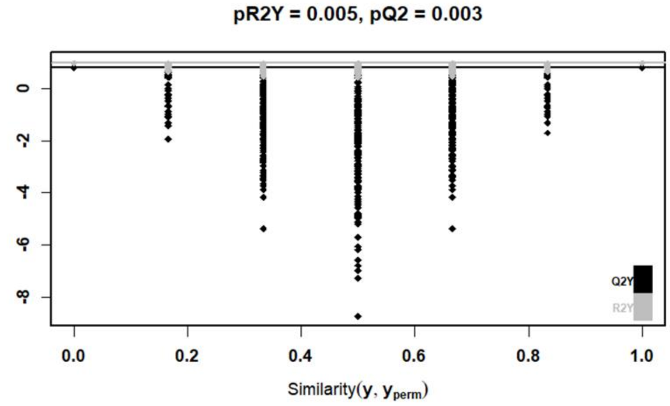
pR2Y = 0.005, pQ2 = 0.003
Q2Y
R2Y
Similarity(y, yperm)

Figure S4: PCA based on biochemical injury markers and BA pool in HOPE and SCS group.

Panel A exhibits PCA based on biochemical data alone. PCA scores with 95% of the variance explained on the first component and 5% on the second component.

Panel B exhibits PCA analysis based on biochemical data and bile acids. PCA scores with 94% of the variance explained on the first component and 5% on the second component.

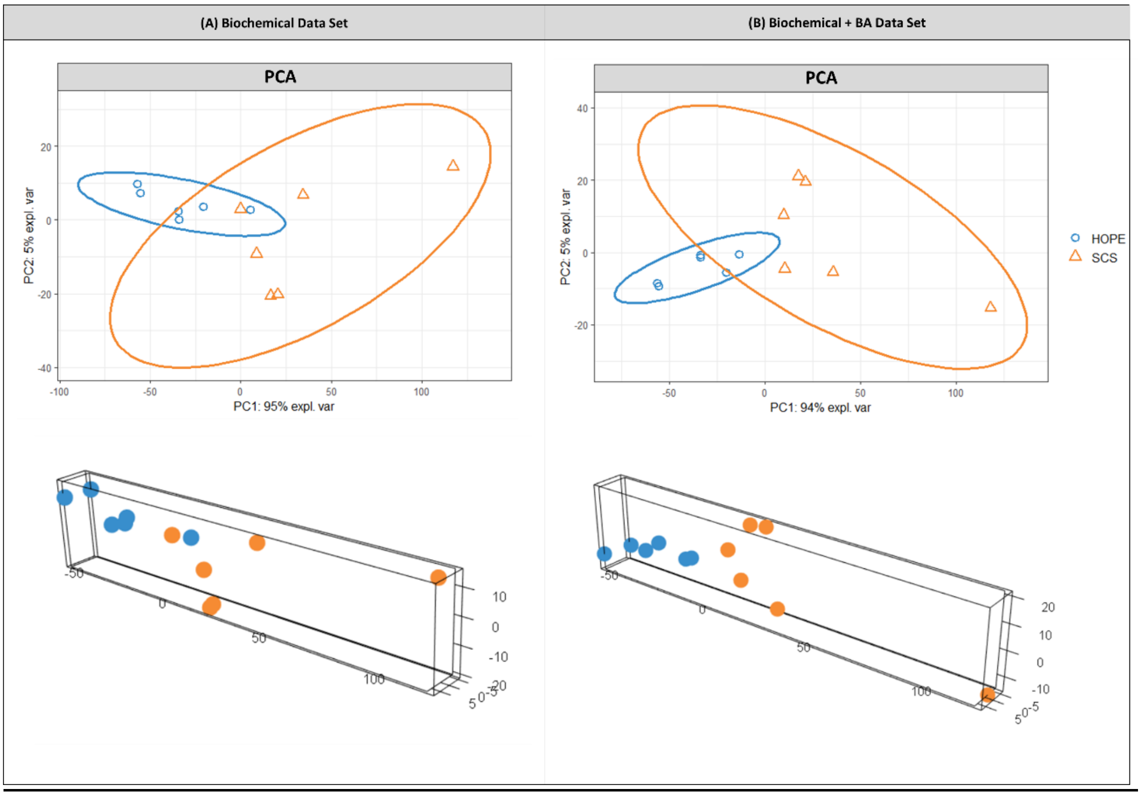

Supplement: Supplementary file 1 — Supplementary Information. [file 41598_2024_52504_MOESM1_ESM.pdf]
